# Evaluation of the implementation of a patient portal pilot intervention among people with type 2 diabetes at community health centers

**DOI:** 10.3389/fcdhc.2025.1689830

**Published:** 2026-01-06

**Authors:** Julie Wagner, Samuel Akyirem, Joanna Lipson, Helen NC Chen, Robin Whittemore

**Affiliations:** 1 Behavioral Sciences and Community Health, UConn Health, Farmington, CT, United States; 2 Yale School of Nursing, West Haven, CT, United States; 3 Department of Epidemiology, Regenstrief Institute, Center for Biomedical Informatics, Indiana University Richard M. Fairbanks School of Public Health, Indianapolis, IN, United States; 4 Division of Nursing Science, School of Nursing, Rutgers University, New Brunswick, NJ, United States; 5 Department of Family Medicine and Community Health, Rutgers Robert Wood Johnson Medical School, New Brunswick, NJ, United States

**Keywords:** diabetes, patient portal, implementation, community health center, health related social needs

## Abstract

**Introduction:**

A pilot study of a multilevel, 6-month intervention (MAP) designed to increase patient portal use among patients with type 2 diabetes at community health centers (CHCs) showed promising results. The aim of this implementation analysis is to (1) describe the nurse–patient interactions and documentation of care during MAP, (2) report MAP implementation successes and challenges, and (3) describe participants’ use of other online health resources.

**Methods:**

Data were collected from MAP nurses (*n* = 3) and participants (*n* = 22).

**Results:**

The content of portal messages between nurses and participants was educational and supportive. Numerous health-related social needs that influence participant diabetes self-management were identified, many of which were handled with relevant referrals. Participant-reported challenges changed over time, with technical barriers decreasing and competing demands increasing. Participants increased their use of online resources for health improvement.

**Discussion:**

Addressing implementation challenges may allow the expansion of programs like MAP in CHCs and ultimately improve diabetes outcomes.

**Clinical Trial Registration:**

clinicaltrials.gov, identifier NCT05180721.

## Introduction

Diabetes self-management is demanding and can be particularly challenging for patients with limited healthcare and financial resources (1). Diabetes self-management education and support (DSMES) encourages health behaviors, including glucose self-monitoring, taking medication, healthy coping, healthy eating, and physical activity. These self-management behaviors have been shown to improve glycemic control, psychosocial outcomes, and prevent or delay disabling and life-threatening complications (2).

Patient portals hold promise for delivering DSMES. Patient portals are digital platforms that typically provide the ability to message providers, schedule appointments, request medication refills, and view personal health information, such as medications, laboratory results, and visit summaries (3). Among people with diabetes, use of patient portals is associated with higher rates of complication screening (4), better control of glycemia, cholesterol, and blood pressure (5), and more frequent data updates in the medical record (6). However, those with low income, low literacy, and low educational attainment are less likely to use portals (7, 8). People who identify as Black, Hispanic, Asian, and those who are Spanish-speaking have lower portal use compared to their White and English-speaking counterparts (8–14).

In the USA, where there are wide disparities in access to healthcare, community health centers (CHCs) are safety-net healthcare centers that provide comprehensive, high-quality primary care regardless of ability to pay or insurance status. Each year, CHCs provide care to over 20 million people with low income, 17 million people of color, 15 million enrollees in Medicaid, and nearly 6 million people who are uninsured (15). In 2024, 94% of CHCs reported that patient access to broadband or technology was a challenge (16). Access is just one of many health-related social needs (HRSN) that are not uncommon among people receiving care at CHCs. According to the Centers for Medicare and Medicaid, HRSNs are individuals’ social and economic needs that affect their ability to maintain their health and well-being (17). HRSNs include economic needs, such as lost employment, as well as interpersonal needs, such as social isolation. HRSNs can be understood as the individual-level experience of social determinants (or drivers) of health. Uptake of patient portals in CHCs lags, especially among individuals with limited English proficiency (13). Few interventions to increase portal use have focused on CHCs (18).

Using qualitative findings from stakeholders (19), a multilevel intervention to improve access to patient portals (MAP) was designed for CHCs to activate and increase portal engagement in patients with type 2 diabetes (T2D) (NCT05180721). MAP was based on the WHO Health Equity framework (20), which considers material circumstances, psychosocial factors, behavioral and biological factors, and the healthcare system. MAP components included Internet access for the study duration, a study-provided tablet (that participants kept at the end of the study), and training from community health workers to use the tablet and portal. A CHW is a frontline public health worker who is a trusted member of, and has an unusually close understanding of, the community served and often works as a bridge between health/social services and the community. MAP also included individual DSMES delivered by a clinic nurse through the portal. We previously reported that the MAP pilot study (*n* = 22) was feasible and acceptable with high levels of treatment fidelity, patient satisfaction, and a good working relationship between participants and nurses, as reported by participants (21). MAP produced 100% portal activation and high engagement with 98% of participants logging in at least twice per month in the first 3 months and 76% between 3 and 6 months. Diabetes distress was assessed with the 20-item Problem Areas in Diabetes scale (PAID) (22) (e.g., “feeling overwhelmed by your diabetes”) with response options on a 5-point Likert scale from 0 = not a problem to 4 = serious problem. Diabetes distress decreased from baseline to 3 months; it continued to decrease from 3 to 6 months. The proportion of clinically elevated diabetes distress (i.e., PAID > 40) was 50% (11/22) at baseline, and it decreased to 36.4% (8/22) at 3 months (*p* = 0.20) and 22.7% (5/22) at 6 months (*p* = 0.03). We also reported that MAP improved participants’ technology confidence, digital health literacy, and DSM self-efficacy.

To refine MAP and expand adoption in CHCs, a better understanding of clinic implementation is needed. Therefore, we conducted an implementation analysis of the aforementioned pilot study, which augments our previous report of primary outcomes. The updated Consolidated Framework for Implementation Research (CFIR) guides qualitative and quantitative assessment of implementation efforts (23). CFIR broadly distinguishes three important constituents: innovation recipients (e.g., patients receiving treatment), deliverers (e.g., clinicians who deliver the treatment), and key decision makers (e.g., clinic directors). It also distinguishes between actual outcomes (perceptions of success or failure) and anticipated outcomes (likelihood of future success or failure). The model also includes the role of determinants that explain outcomes. Focusing on actual outcomes and determinants from recipients and deliverers, the purpose of this analysis is to (1) describe the nurse–patient interactions and documentation of care during MAP, (2) report MAP implementation successes and challenges, (3) describe participants’ use of other online health resources and their intentions for future portal use, and, (4) make recommendations for broader implementation and more rigorous testing of MAP.

## Materials and methods

### Overview

The Yale University Institutional Review Board approved this study. MAP was piloted using a within-subjects, pre–postdesign in 22 adults with T2D who were portal-naïve. Participants were recruited from primary care provider (PCP) panels at two CHCs in Connecticut, a small, densely populated state with pronounced diabetes disparities, located in the northeast USA. Inclusion criteria were as follows: age 21–65 years; diagnosed with T2D for > 6 months; most recent A1C > 7.5%; no use of the patient portal in the past year; no intention to change clinics within 6 months; and self-reported ability to read in English or Spanish. Exclusion criteria were current gestational diabetes or cognitive impairment (24). If eligible and interested, informed consent was obtained. Recruitment and data collection took place from May 2023 to July 2024. Assessments were at baseline, 3 months, and 6 months; participants were compensated with a gift card for $40 at baseline and 3 months and $60 at the 6-month timepoint.

### Intervention

As previously reported, all participants received standard T2D care at their CHC (25). Participants met with CHWs and nurses, whose training has been described previously (26). First, CHWs met individually with participants to assess health-related social needs and make referrals, e.g., the Supplemental Nutrition Assistance Program (SNAP or “food stamps”). CHWs then provided each participant with a tablet, delivered individualized training on its use, installed the clinic portal application, and introduced participants to portal navigation. CHWs remained available to address questions and technology-related challenges. All CHWs were bilingual and bicultural, and in a few cases, research staff provided additional technical support.

Next, a nurse met individually with each participant to build rapport, assess DSM behaviors, and collaboratively develop a DSMES plan. Following this, nurses (*n* = 3) were instructed to message patients through the portal at least twice weekly for the first 3 months and then weekly for the next 3 months. In these individualized messages, nurses assessed progress with the DSM plan; provided education, support, and encouragement; and shared electronic health education resources via the portal. Each month, nurses sent written summaries to each participant’s PCP regarding health status and patient progress/challenges. The nurses had extensive experience (12–44 years in practice), and one was bilingual in Spanish/English.

### Measures and data collection

#### Demographics

At baseline, participants reported age, gender, race/ethnicity, educational attainment, income, insurance status, preferred language (English or Spanish), and frequency of requiring assistance reading health information (never/rarely/sometimes/often/always). Research assistants read assessment questions and response options aloud to participants and entered their responses into REDCap™ (27), an electronic data capture application.

#### Portal messages

Information technology (IT) staff at each clinic extracted the content of portal messages exchanged between MAP nurses and participants. The messages were analyzed using a rapid content analysis method (28). A codebook was developed after coding three transcripts, based on the intervention protocol, to identify overarching message content from both the nurses and participants. Two research team members (RW, JL) independently coded and/or reviewed the transcripts and resolved discrepancies through discussion, using Microsoft Word comments to track codes. To ensure methodological rigor, we re-examined transcripts to confirm that the coding process accurately reflected the data and employed a collaborative approach to finalize the analysis.

#### Nurse summaries to primary care providers

Nurses documented a monthly summary of patient portal communications for each participant’s PCP, including medical, educational, and psychosocial updates, so that providers were aware of factors influencing DSM. Any psychological or social issues were extracted and coded by two research team members (HC, RW) using the rapid content analysis method described earlier.

#### Nurse-reported protocol implementation

The co-PIs (RW, JW) conducted semistructured interviews with nurses at the conclusion of the study. Interview guides were collaboratively developed by the research team to assess implementation processes considered critical in many implementation models (29). Nurses were asked to describe their overall experience with implementing the MAP protocol, the adequacy of training, challenges encountered, successes achieved, and recommendations for the future. They also provided brief written responses to each question in the interview guide. Data were coded and analyzed using the rapid content analysis method described earlier, with the interview guide as the coding framework, by two research team members (JL, RW).

#### Participant-reported challenges to portal use

At 3 months, CHWs and/or research staff assessed participant challenges, such as forgotten passwords, to provide troubleshooting or additional training on portal use. Participants were also asked about literacy-related challenges, including “Are you able to read and understand the message?”, “Do you have any concerns about your writing or spelling skills?” (at 3 months), and “Were there ever times you felt it would take too long to write your concern to your nurse?” (at 6 months).

#### Participant-reported use of online resources

At 3 and 6 months, participants were asked whether they had used the tablet to access online resources to “improve your health in other ways”, such as “downloading health apps like a glucose tracker or calorie counter”.

#### Participant impressions of the portal and intentions for future use

At 6 months, participants answered open-ended questions about what they liked about using the patient portal, how it was helpful for their diabetes care, and whether they planned to continue using the portal after completing the study.

## Results

Of the 47 recruits approached, 26 provided informed consent, and 23 received the intervention, with one lost to follow-up, resulting in 22 participants included in the analysis. The sample had a mean (± SD) age of 56.3 (± 10.9) years; 73% (*n* = 16) were women, and most were Latino/Hispanic (77%, *n* = 17). The mean duration of diabetes was 11.7 (± 9.1) years, and the mean HbA1c was 9.0 (± 1.5). For participant characteristics, see **Table 1**.

**Table 1:** Participant characteristics at baseline.

Variables	% (N) or mean (SD)
Participants recruited from the two clinic sites and the clinic’s patient portal platform	
Clinic 1 (Healow patient portal platform) (%)	68% (15)
Clinic 2 (MyChart patient portal platform) (%)	32% (7)
Age in years	56.32 (10.93)
Sex	
Women (%)	73% (16)
Married/Partnered (vs. not)	55% (12)
Ethnicity	
% Hispanic/Latino	77% (17)
Race	
White (%)	45% (10)
Black (%)	18% (4)
Other (%)	36% (8; of them, 6 reported Latino/Hispanic)
Employment	
Working part/full time (vs. others) (%)	36% (8)
Education	
Less than high school graduate (%)	59% (13)
Annual income	
< $40,000 (%)	86% (19)
Preferred language	
Spanish (%)	64% (14)
Able to converse in English (%)	45% (10)
Assistance with reading health information	
Never (%)	50% (11)
Rarely/Sometimes (%)	32% (7)
Often/Always (%)	18% (4)
Health insurance	
Insured (%)	45% (10)
Home Internet?	
No or unsure (%)	15% (3)
Home cell phone data stability	
Unsure	15% (3)
Fair	27% (6)
Good	32% (7)
Very good	27% (6)

### Portal messages

Portal messages were coded separately for nurse-to-participant messages and participant-to-nurse messages (**Tables 2**, **3**, respectively). Unique nurse-to-participant messages included checking in; following up on referrals, medical appointments, or diabetes care recommendations; providing diabetes-related education; and offering support, motivation, or encouragement. Nurses also referenced in-person appointments or phone calls and, on occasion, communicated directly with the participant’s primary care provider regarding upcoming appointments.

**Table 2:** Nurse to participant portal message interaction.

Type of message	Examples
Check-in	“How can I best support you with your diabetes this week?” “Hello, I was wondering if the [continuous glucose monitor] is working for you. If so, what numbers are you seeing? Do you think your average blood sugar level is still around 250? Remember, your goal was to reduce your HgbA1c below 9.0%.”
F/U for PCP appts, referrals, med changes, hospitalizations, etc.	“I understand that Maria scheduled an eye appointment for you. Please let me know if you were able to attend this appointment.” “I spoke with the pharmacy, and they will call you today to arrange the delivery of the new blood test machine. Please let me know if you do not receive it and if you have any problems with the medications they send.”
Education	“Below are some links to plant-based diet resources that may also be of interest.” “This week I am sending along a link in Spanish about 4 Steps to Control your diabetes for life.” “Here is some information in Spanish about food during this time when prices are so high.”
Support, encouragement, motivation	“Wow, your A1C really came down. That’s amazing! Keep up the good work.” “I can see that you are working very hard to stay as healthy as possible while also dealing with the stressors in your life. Keep up the good work!”
Personal connection (sharing a little about self, platitudes)	“I am so, so sorry to hear about the loss of your mother. How are you holding up? I wish you peace and comfort, understandably its a difficult time right now.” “I hope everything is going well with your new job.”
Communication directly to PCP	“Patient stopped by the RN office yesterday. States she saw the nutritionist at NH who she said wants her to complete labs with PCP for her “thyroid and hormone levels”. Printed out consult note for your review. Patient wants to discuss increasing medications or trying new medications for her diabetes. She is very concerned about her recent HgbAlc as well as reported 4 lbs weight gain lately. Patient has follow up appointment scheduled for 9/12.” “I called and spoke to patient. I scheduled her for a televisit on 8/24 at 2:20 pm. She wants to discuss labs for thyroid levels. Also states nutritionist recommended sleep study due to her poor sleep. Wants to discuss.”
Reference to in-person/phone calls	“I will see you Saturday. It will be nice to meet you in person.” “Tuesday morning is good for a phone call.”

**Table 3:** Participant to nurse patient portal interaction.

Type of message	Examples
Updates	“I’m spoke with Doctor X last Saturday and my AlC was 10.” “I’m still struggling with eating too many sweets and I’m going to try my hardest to minimize them or cut them out entirely for the next couple weeks.”
Confirmation	“I will take your recommendation into account. It’s good to know the possible risks of diabetes.” “Thank you very much, I have seen your message.”
Help with appointment	“I have appointment for my elbow surgery July 21 and they say I have to meet with doctor X before. So can you help me make appointment for her. I have appointment July 29 but it’s too late.” “Could you arrange a visit with Dr. X?”
Questions	“I know Dr. X was thinking about switching my medication but we weren’t able to get my A1C last time…. ls switching medications dependent on what my A1C is?” “My main concern with my Diabetes is that I continue to get higher readings in the morning … I’d like a little more information on the dawn phenomenon…”
Personal connection	“It’s beautiful weather! How was your long weekend?” “Nice to hear from you!”
Appreciation	“Thank you so much about all information. You are the best!!!” “Thanks so much for the links. I’ve visited each of the websites and ended up ordering a cookbook [for diabetes]”

Unique participant-to-nurse messages included updates on diabetes or life challenges affecting DSM, confirmation of information received from the nurse, requests for assistance with scheduling appointments, questions about diabetes or other health concerns, and expressions of appreciation. Nurses and participants also exchanged messages of personal connection that supported relationship-building, such as vacation plans or sending well wishes during difficult situations.

### Nurse summaries to PCPs

In their monthly written summaries to PCPs, nurses documented factors that patients reported as affecting their attention to DSM. Family stress (e.g., caregiving for a disabled child, death in the family) was reported by 50% of participants, financial concerns (e.g., housing instability, unemployment) by 36%, medical issues (e.g., pain, hospitalization) by 27%, and psychological issues (e.g., depression, substance use, stress) by 23%. Many participants reported several such factors over the 6-month study duration, and nurses made referrals for 50% of participants to medical specialists (e.g., endocrinology, podiatry), behavioral health services, or social services. Per protocol, nurses also reported standard medical information to PCPs, such as glycemic control and medications.

### Nurse-reported implementation of the MAP intervention protocol

Overall, nurses reported that their training on the protocol was adequate and that its implementation was generally positive, particularly with patients who actively engaged via the portal. One nurse reported that getting patients engaged in care “is a victory for all”. However, several challenges to implementing the MAP intervention were identified. A primary concern was the lack of participant responses or, in some cases, very brief responses such as “si” and “hola”. This lack of engagement was frustrating for nurses, who were unsure whether the issue was technical, literacy-related, or personal, and consequently uncertain how to respond. Nurses also faced difficulties due to the portal configuration at one clinic, which made it hard to track messages that participants had not opened. Language barriers posed additional challenges, as monolingual English-speaking nurses relied on online translation tools to read and write messages in Spanish, which could hinder accurate communication. Uploading educational resources to the portal was cumbersome because nurses had to manually add links or PDFs for patient materials. Finally, portal or clinic constraints on messaging from patient to PCP complicated communication in some cases, as not all providers accepted messages from patients. The patient portal included cumbersome and error-prone features for transmitting glucose data to clinicians. Additionally, all nurses reported that the protocol’s messaging schedule was too frequent and expressed a preference for a less structured approach.

### Participant-reported challenges to portal use

During the first 3 months, participants faced challenges accessing the portal, such as password issues, which were handled by CHWs or research staff. By 3- and 6-month assessments, no participants reported difficulties using the tablet or signing into the portal. Regarding literacy, 13.6% (*n* = 3) indicated they could not read or understand the messages from the nurse, 13.6% (*n* = 3) expressed concerns about their writing or spelling skills, and 18.2% (*n* = 4) felt that composing their concerns in a portal message would take too long.

As shown in **Table 4**, the number of participants reporting “not being able to respond to nurse messages” and “not having the tablet on hand” decreased to zero from 3 to 6 months. However, the number of participants endorsing other barriers increased slightly over the same period, including lack of time, forgetting, concerns about privacy, and uncertainty about what to say. Qualitative comments from participants identified additional barriers, such as low educational attainment, low reading skills, not knowing how to respond, preference for face-to-face communication, the portal training occurring too long ago, messages not going through, unstable connection, slow internet, and using alternative devices (e.g., smartphone) for daily use that did not have the portal app installed. One Spanish-speaking participant relied on her adult daughter to access the portal and message on her behalf.

**Table 4:** Patient-reported challenges to using the patient portal for messaging.

Questions on portal use challenges	Participant Response 3 months			Participant Response 6 months		
	Yes	No	% with challenge	Yes	No	% with challenge
1 Are you able to … use a tablet, log in, type a message? (Circle any yes)	100% (22)	0	0	100% (22)	0	0
2. Are you able to see the messages from your nurse?	100% (22)	0	0	100% (22)	0	0
3. Are you able to respond to messages from your nurse?	82% (18)	18% (4)	18% (4)	100% (22)	0	0
4. Are you able to take time out of your day to sign in, review messages, and respond to messages?	100% (22)	0	0	91% (20)	9% (2)	9% (2)
5. Do you sometimes forget or have other things on your mind?	0	100% (22)	0	41% (9)	59% (13)	41% (9)
6. Do you have difficulty because you don’t have a tablet available to check it?	36% (8)	64% (14)	36 (8)	23% (5)	77% (17)	23% (5)
7. Do you have any concerns about privacy, who will read messages?	18% (4)	82% (18)	18% (4)	23 (5)	77% (17)	23% (5)
8. Do you ever feel like you are not sure what to say?	27% (6)	73% (16)	27% (6)	36% (8)	64% (14)	36% (8)
9. Do you ever feel like you don’t have any problems with your diabetes management to write about?	18% (4)	82% (18)	18% (4)	23% (5)	77% (17)	23 (5)

### Participant-reported use of online resources

**Figure 1:**
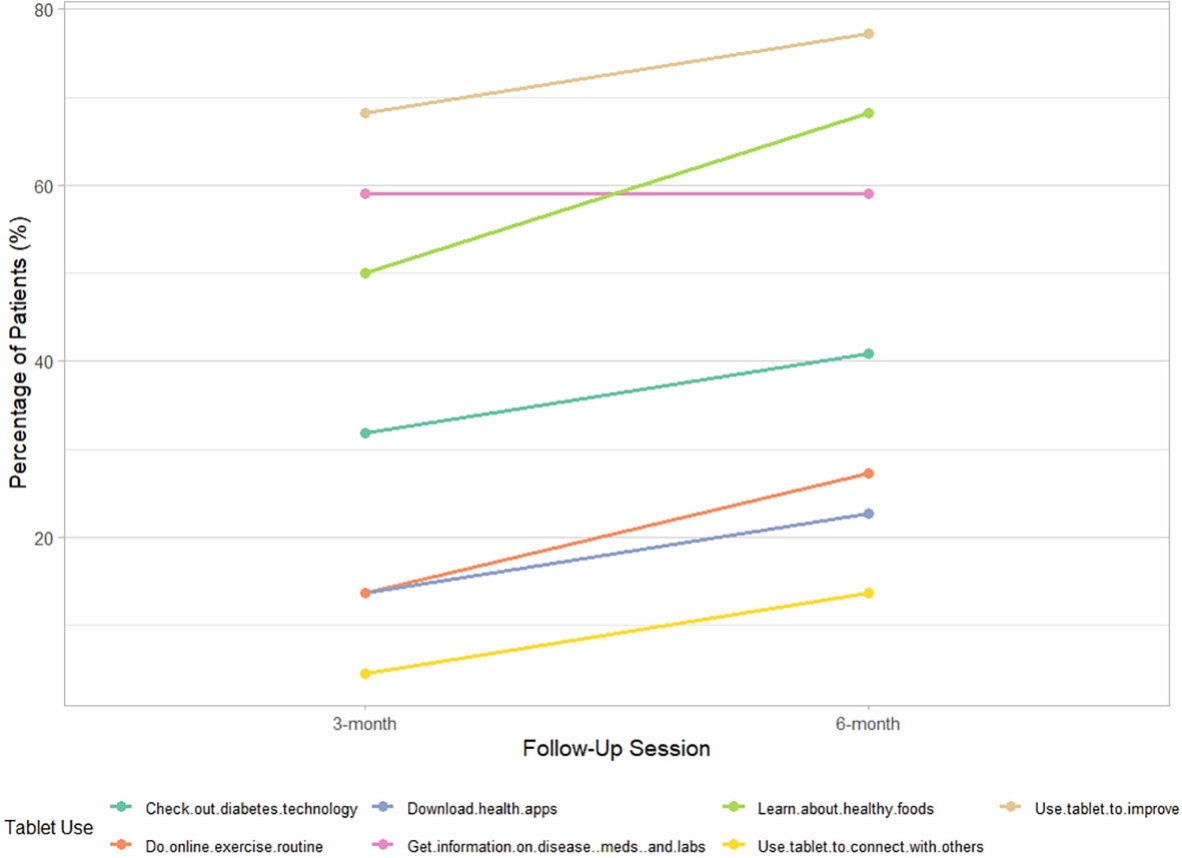
Tablet usage patterns over time in the patient portal intervention.

As can be seen in **Figure 1**, between 3 and 6 months, there was a noticeable increase in the percentage of participants reporting use of the tablet to access online resources for DSM.

### Participant impressions of the portal and intentions for future use

In response to open-ended questions about what participants found helpful in the MAP program at 6 months, 86% cited messaging/communication with the nurse; 64% noted access to lab results, medications, and visit summaries; 59% mentioned scheduling appointments/receiving appointment reminders; and 32% reported gaining a better understanding of their diabetes. Participant comments included appreciation for “the worry and care of the nurse”, “having a team that cares and wants to help with your diabetes journey”, and “the information about diabetes and what to eat”. At 6 months, 100% of participants felt the portal was helpful for their diabetes care, and all indicated that they would continue using the portal for diabetes management after the study ended.

## Discussion

In this analysis, we report on the implementation of the MAP protocol, drawing on elements of the CFIR that were relevant to our pilot study. We have demonstrated that nurses in CHCs can take a more active role in managing T2D through the patient portal by checking in with patients, following up on recent or missed medical appointments, and providing diabetes education, support, and encouragement. This ongoing virtual nurse–patient communication helps establish relationships and allows patients to discuss personal life stressors, medical issues, and psychological factors that affect DSM, with relevant information communicated to the patient’s PCP. Patients reported that nurse communication was the most helpful aspect of using the patient portal, followed by accessing medical records and managing appointments, highlighting the value of personalized attention.

However, to successfully increase patient portal use among adults receiving care in CHCs, several implementation challenges must be addressed. Nurses reported difficulties such as limited participant responses to messages, language barriers, and difficulty uploading personalized diabetes education resources. Participants experienced technical issues when accessing the patient portal, requiring support beyond the CHW training, particularly during the first 3 months of protocol implementation. Over time, some participants continued to face challenges in accessing messages due to limited time, forgetfulness, or not having the patient portal app installed on their cell phones.

The MAP protocol directed nurses to send messages focused on check-ins, education, motivation, and self-management support. Analysis of message content supports treatment fidelity. Other nurse-to-participant messages included follow-up on medical care appointments and DSM recommendations. Participants asked questions, although less frequently than they responded to nurse messages. They also sent messages expressing personal connection and frequently showed appreciation for the nurse. Approximately one-third of participants reported having a better understanding of their diabetes. These findings are consistent with studies showing that patient portals can improve the quality of patient–provider communication (30–32) and facilitate lifestyle changes (33, 34). However, engaging patients via the patient portal can be challenging, particularly for non-English-speaking adults or those with low health literacy. Voice-activated messaging may be a useful feature in the future. Portals with video capability may also be a benefit to patients who are amenable to using them.

The content of the nurse’s written documentation to PCPs highlights the numerous HRSNs that were reported by participants to nurses. These data demonstrate the complexity of the lives of patients who access healthcare at CHCs and their resilience in nonetheless engaging with DSM. In 2024, between one-half and three-fourths of CHCs screened patients for HRSNs, including needs regarding housing, transportation, food, utilities, and interpersonal safety (16). Portal interventions like MAP may provide a second safety net, i.e., an opportunity for patients to disclose difficult life circumstances to nurses, who can play an important role in care coordination (35).

HRSNs also posed common challenges to messaging, including low literacy, low education, lack of “knowing what to say”, or not perceiving any issues with their diabetes. These findings highlight the importance of interventions like MAP employing strategies tailored for low-literacy communication (36). Utilizing a portal proxy (friend, family member) may also be beneficial, as observed with at least one MAP participant. Supporting this approach, a study of people with T2D > 50 years of age found that secure messaging via portal proxies increased messaging frequency and enhanced patient–clinician telecommunication (37).

Challenges with messaging that increased over time were related to factors such as competing priorities. This highlights the benefits of portal communication, offering frequent and ongoing opportunities to identify and address challenges for patients with complex social needs. Technical issues with messaging, in contrast, tended to occur early and were generally resolved with support. It is essential to have a designated person in CHCs who is fluent in the languages of clinic patients to help address portal-related technical challenges. Low technology confidence remains a barrier to portal use (18, 19); as previously reported, MAP improved patients’ technology confidence (21). Indeed, many patients independently used the tablets to access online health resources. Device ownership is associated with health literacy, and individuals with greater access to technological devices, such as smartphones/tablets, are more likely to seek health-related information online (38).

Research indicates that patient portal use improves health outcomes by enhancing engagement in care and patient–provider communication. For example, people with diabetes report that reading physician notes in the portal is extremely important for managing their health (39). Although further research is needed, our findings suggest that additional mechanisms for improved health outcomes may include fostering interpersonal connection, checking in, following up on medical appointments and referrals, and providing education and support. For socially and medically complex patient populations, portals offer an additional method of communication and access to personalized, accurate health information. Although we do not have data to speak to this, we hypothesize that automated messages from the clinic would not produce the same effect. We propose that the personal connection and support conveyed through portal messaging actively engage patients in their own DSM. Evidence indicates that targeted portal messages are more effective than generic messages (40). While personalized nurse-to-patient messages, such as those delivered in MAP, are more resource-intensive than generic communications, they may be necessary—and potentially cost-effective—for patients facing significant barriers to DSM, such as those receiving care in CHCs. Supporting this, data from patients with T2D show that even a single non-face-to-face contact for chronic care management led to reduced emergent care, increased routine care, and improved health outcomes compared with patients without remote care management (41).

Limitations of this study include a small sample size, with only three nurses and 22 patients, conducted in a single geographic location. The study employed a single-group design without randomization to treatment groups. Not all clinics have CHWs or nurses available to support interventions like MAP. Demand characteristics may have influenced reports from both nurses and participants. Additionally, retention strategies—such as providing gift vouchers and allowing participants to keep the tablet after the study—could have introduced bias in the observed low attrition.

The findings suggest several key recommendations to support the successful adoption of patient portal programs in CHCs. Screening for literacy and health-related social needs is essential to ensure patients are ready to use the portal. Patients may report unmet HRSNs that affect DSM throughout the course of clinical care, not only at the initial assessment. Allowing patients to include a portal proxy can be beneficial for those who need additional support. Designated clinic personnel, such as CHWs, patient navigators, or medical assistants, should be available to provide flexible portal training and technical support, which can enhance engagement over time. Tailoring the frequency of portal messages to each patient’s clinical needs may improve efficiency. Finally, nurse case management for patients with T2D can integrate ongoing DSMES via the portal, building on relationships initially established in person at the clinic (42).

As patients with T2D accessing care in CHCs increasingly gain access to smartphones and the Internet in the community (e.g., coffee shops, libraries), interventions like MAP have the potential to enhance portal use, patient–provider communication, and health outcomes. However, careful consideration is needed to address implementation challenges, including allocating staff for technology training and support, establishing guidelines for nurse case management and DSME via the portal, and determining reimbursement strategies for staff time on portal-based programs. Future research using models such as the CFIR may help guide these efforts.

## Data Availability

The raw data supporting the conclusions of this article will be made available by the authors, without undue reservation.
